# HPV and cervical cancer: screening or vaccination?

**DOI:** 10.1038/sj.bjc.6604146

**Published:** 2008-01-08

**Authors:** F X Bosch, X Castellsagué, S de Sanjosé

**Affiliations:** 1 Cancer Epidemiology and Registration Unit, IDIBELL, Institut Català d'Oncologia, L'Hospitalet del Llobregat, Barcelona, Spain

**Keywords:** cervical cancer, HPV vaccines, HPV screening

## Abstract

Following the demonstration of the superior validity of human papillomavirus (HPV) tests in screening for cervical cancer and the arrival of highly efficacious HPV 16 and 18 vaccines, cervical cancer prevention enters a time of sustainable introduction in developing countries. Multidisciplinary efforts and novel protocols are being developed, and challenging situations are being faced to make cervical cancer, still the number two cancer in women worldwide, an eradicable condition.

The provocative title proposed by the editors of the *British Journal of Cancer* could have at first sight, a quick and intuitive answer: we will need both. We need human papillomavirus (HPV) vaccines to significantly reduce the health care burden currently required for cervical cancer prevention, and we need screening because of the limitations of current HPV vaccines both in their lack of therapeutic effect (thus not protecting women with an ongoing neoplastic processes) and in their limited number of HPV types (thus leaving to evolve some 25–30% of cervical cancer cases related to HPV types other than 16 or 18). However, the answer only applies in scenarios in which screening is already developed and reasonably efficient. In populations without adequate screening, one could equally argue that HPV vaccines at affordable prices are the only realistic option. While these arrive, more efficient screening schemes, for example with low-cost HPV tests, requiring fewer visits or strategies involving rapid intervention like ‘screen and treat’ protocols, remain the only option for currently living adult women. Moreover, should polyvalent vaccines (including some five–eight HPV types) result in extending protection against more than 90%+ of the oncogenic HPV types, vaccination alone would be the answer for both scenarios. Thus, a complex answer to an apparent straightforward question.

## PHASE III HPV VACCINATION TRIALS

With the publication of the key short-term results of the two major Phase III trials of HPV vaccines, the perspective of tackling cervical cancer prevention with vaccination has been unambiguously open. While recognising the limitations of the still moderate (5–6 years) follow-up in a few tens of thousand young women, two vaccines to date have shown high efficacy, safety, immunogenicity, long-term duration of protection and a strong suggestions of induction of immune memory (Harper *et al*, 2006; Garland *et al*, 2007; Paavonen *et al*, 2007; The Future II Study Group, 2007).

A number of clinically relevant issues remain to be fully described, including the magnitude and the HPV spectrum included in the cross protection effect, and the long-term effects of each of the HPV vaccines on cancer protection and safety. However, to solve these questions, it is unavoidable to continue the studies for additional follow-up time and the organisation of large Phase IV studies, some of which are already in place.

The currently available vaccines offer full protection to HPV 16- and 18-naïve women for these two HPV types that cause an estimated 70% of cervical cancer and a slightly lower fraction of its precursors. Once the cross protection impact is fully described and the geographical variation of the HPV types in cervical cancer is better known, these estimates will likely increase in some areas to perhaps 75–80%. Human papillomavirus 16 and 18 account for a higher proportion of cervical adenocarcinomas, in the range of 80–85% (Castellsague *et al*, 2006), the histological subgroup that more easily escape detection by cytology-based screening practices.

Table 1 shows a selection of the established qualitative findings thus far on both HPV vaccines. Notice that the *Gardasil*® program has already communicated results of its 2-year follow-up in the per protocol population, evaluating efficacy in HPV DNA-naive women at study entry. In contrast, the *Cervarix*® Phase III program has only published a prespecified, interim report including the efficacy analyses on the intention-to-treat population, thus including women with HPV and low squamous intraepithelial lesions (LSIL) at recruitment and a follow-up time of 15 months.

## NEW CENTRE OF GRAVITY OF CERVICAL CANCER PREVENTION: HPV VACCINE IMPLEMENTATION AND ACHIEVEMENT OF VACCINATION COVERAGE

**Figure 1:**
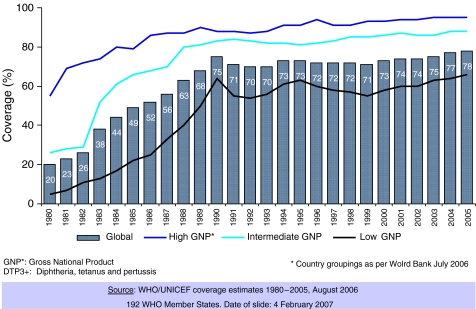
Expanded Program of Immunization 1980–2005 DTP3+ coverage by level of development.

On a worldwide scale, the centre of gravity of cervical cancer prevention has shifted towards a more established mindset in the field of vaccinology such as ensuring production and access to HPV vaccines and devising the logistics to ensure wide coverage of the target populations. The Expanded Program of Immunization (EPI) is probably one of the most successful public health efforts in place. Figure 1 shows the estimated worldwide coverage of infants with three doses of DTP (diphtheria, tetanus and pertussis). Globally, the programme ensures vaccination to 70–75% of the children, and the socioeconomic gap between the wealthiest and the poorest countries is in the order of 25–30%, while the gap in terms for example of Gross National Product (GNP) and other health indicators is several fold higher (Figure 1). Vaccination of infants is thus feasible in developing countries and vaccination programmes such as the EPI have developed and maintained in place a considerable infrastructure and logistics network. Vaccination of adolescent in contrast might represent the greatest challenge in many developing populations, and several explanatory and demonstration projects are now underway (http://www.path.org/news/).

Public health researchers thus have an important task ahead, namely to organise the trials that will guide how to best evaluate and, if appropriate, to integrate HPV vaccines within the structure of the EPI. These will certainly take time and should address issues such as routes of administration (i.e., injection *vs* oral), number of doses required (as well as antigen amount per dose), immune response and duration of protection when administered in these age groups, and tolerability and safety when administered with other EPI vaccines. The need and the opportunity of a booster dose latter in life (i.e., at the time of presenting to the EPI stations with their first baby) should also be evaluated.

Alternatives such as the deployment of a platform for vaccination of adolescents (for example, based on schools) could be considered in some countries with adequate infrastructure.

## THE SIZE OF THE PROBLEM AND THE SIZE OF THE SOLUTION

**Figure 2:**
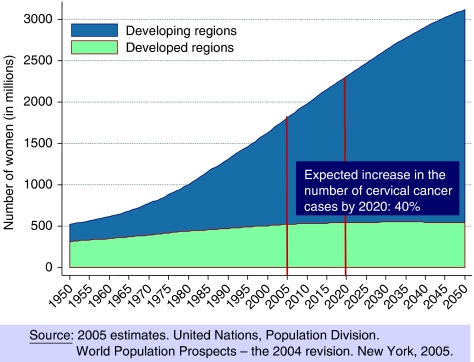
World population prospects for women ⩾15 years.

The population of women in the world continues to increase. Figure 2 shows the predictions for the period 2000–2050 and clearly indicates the expected growth of the female population (ages 15+ years) in developing countries and a stable prediction in the developed countries. By age groups, these estimates reflect for girls 10–14 years and women 15–24 years, a plateau in the developing countries and a decrease in the younger populations in developed countries, largely in the age groups 15–24 years. With these population estimates, largely attributable to the increased life expectancy in women in developing countries, the International Agency for Research on Cancer (IARC) prediction on the number of cases of cervical cancer anticipated by 2020, all other things being equal, is of an increase of 40% globally. The 40% increase in cervical cancer cases is again dramatically driven by socioeconomic status, and countries in Africa, Latin America and Asia are predicted to increase the number of cases by 50–55%. Europe and North America will also experience a modest increase in the number of cases in the order of 6% in Europe and of 23% in Northern America (Ferlay *et al*, 2004).

**Figure 3:**
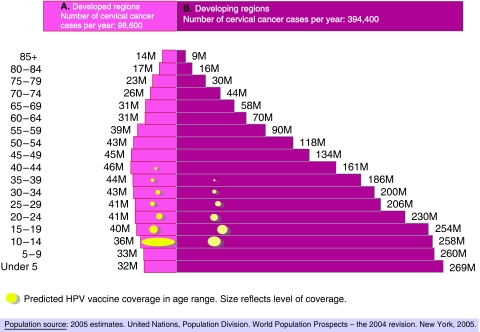
Worldwide female population and a speculative anticipation on the initial introduction of HPV vaccines.

The number of women in any 1-year age cohort aged between 10 and 14 years has been estimated to be close to 60 million (Figure 3). Of these, some 52 millions (87%) live in developing countries. Vaccination of the 5-year preadolescent cohorts aged 10–14 years would require approximately 1 billion doses of HPV vaccine (accounting for a 10% waste). Should a catch-up strategy be put in place, increasing the vaccination target to women 10–25 years would increase the vaccine requirements for the initial vaccination rounds to a target of up to 15 billion doses. There is a clear need to address early in the process the phasing stages of this introduction and, most importantly, to understand with vaccine manufacturers how these quantities can be produced, where are the strategic production countries and the time it will take to put vaccines into their target delivery points. These parameters are needed to anticipate the time scale in which worldwide HPV vaccine introduction will be realistically an achievable goal.

Human papillomavirus vaccines have a cost in 2007 that exceeds the current possibilities of many countries. It is thus anticipated that for some time after introduction, access to vaccination will also reflect the different opportunities related to socioeconomic status. Previous experience with the introduction of the hepatitis B virus vaccine in developing countries has documented that vaccine cost is an essential component of a successful introduction and a determinant of the time to introduction in may parts of the world (Kane *et al*, 2006).

Figure 3 shows a speculative anticipation on the age and social groups that are likely to first receive HPV vaccines in developed and developing countries, outside the organised Phase IV trials and demonstration projects. The predictions are largely based on the initial recommendations to vaccinate preadolescent and adolescent girls (Centers for Disease Control and Prevention, 2007). With this admittedly speculative evidence, one can anticipate that in developed countries (Figure 3A), adolescent groups will receive systematic vaccination in the context of public health-supported programmes and will reach significant coverage within a relatively short time period. Some amount of vaccine will also be administered to a less controlled on-demand population of all ages largely in the private sector. In developing countries (Figure 3B), women are likely to first access HPV vaccines in the private sector across all age groups within the higher socioeconomic strata and only latter, access to vaccine will cascade to wider segments of the adolescent groups and to the general population. The process is likely to get accelerated when vaccine availability and lower pricing generalises. Systematic adolescent vaccination in developing countries will gradually occur in the future, perhaps related to a first vaccination round within the EPI vaccination programme. On these grounds, it is important that additional information on vaccine efficacy and duration of protection when given to infants is made available by researchers (Table 2).

It is thus plausible that, unless a definite and specific massive international intervention occurs, a meaningful introduction of HPV vaccines worldwide will take decades. As a sequitur, for most living women today, screening remains their primary option for cervical cancer prevention. The momentum afforded by the arrival of HPV vaccines should generate an increased awareness and attention to the potential of HPV screening technology as compared to conventional cytology, a scientific evidence that in spite of consistent findings in a number of clinical trials has been only irregularly followed in a significant number of the developed countries.

## ADVANCES IN SCREENING

After 50 years of Pap smears and close to a decade of evaluation of HPV-based screening, a number of considerations on its value and sustainability have been concluded by several major international review parties (International Agency for Research on Cancer, 2005; Arbyn *et al*, 2006; Cuzick *et al*, 2006b; Kitchener *et al*, 2006).

It is important to recognise upfront that in selected countries, reductions of up to 70% in cervical cancer incidence and mortality have been achieved by repeated conventional cytology screening tests. The successful screening programmes are usually centralised and coordinated by the public health systems and require consistent compliance of the population. In developed countries with opportunistic generalised screening and mixed activities in the public and private sectors, some subpopulations are largely over screened and eventually over treated. In many instances, the system is expensive and inefficient. If HPV vaccines are primarily used and/or limited to these populations, the global impact of vaccination on cervical cancer rates will be marginal. If, in addition, current screening practices are maintained, the system will become even more expensive and inefficient.

In the majority of countries in the world, the three-stage conventional screening for cervical cancer (Pap smear, colposcopy/biopsy and treatment) repeated at regular intervals has not been sustainable, and active research is in place to evaluate screening alternatives (Denny *et al*, 2006).

Second, over 15 studies have consistently shown that the use of HPV DNA tests as the primary screening method is significantly more sensitive than cytology-based screening, either conventional or liquid based (International Agency for Research on Cancer, 2005; Cuzick *et al*, 2006a; Ronco *et al*, 2006; Mayrand *et al*, 2007). The summary gain in sensitivity is in the range of 25–35%, and the reasons for it are clearly understood: (a) the presence of HPV DNA is a necessary prerequisite for cervical intraepithelial neoplasia (CIN) 2+; (b) the sensitivity of HPV tests is consistently high (>90%) across different laboratories, with an average level of sophistication in technology; (c) the detection of HPV DNA is independent of the limitations of any human eye observation, as it is the case with colposcopy, cytology and histology; and (d) HPV being often a field infection, the detection of the viral DNA is largely independent of the sampling ability to the point that self-sampling is able to detect HPV DNA at a similar level to that of the professionally collected cervical sample. Additional advantages of HPV screening refer to the automation of the process amenable to high throughput and subsequent lower costs and the recognition that HPV tests will be of great use in the evaluation of vaccination programmes.

The trade off of the high sensitivity of HPV screening is a reduced specificity in the order of 8–12% as compared with cytology, afforded by the high specificity of the cytology test. However, the conventional parameters of sensitivity and specificity traditionally refer to the ability of the tests to detect prevalent disease (cancer or the precursors) at the time of screening. Under this concept, a woman aged 30–35 years or above found positive for a high-risk HPV DNA and normal in the cytological examination would be labelled as a (prevalent) false positive, weighing against specificity. Longitudinal studies have shown, however, that these cases (might be as high as 10% of screening participants) particularly if HPV DNA persists are indeed at higher risk of progression to CIN 2+ within the next decade as compared to women who show normal cytology and are HPV negative (Lorincz and Richart, 2003; Kjaer *et al*, 2006). Clinically, these women are properly identified as part of the high-risk group and candidate to more intense follow-up. Moreover, the 10-year positive predictive value for CIN 2+ is almost entirely due to the HPV tests, largely arguing in favour of using HPV as the stand-alone primary screening test and taking advantage of the higher specificity of cytology/biopsy to triage HPV-positive women (after ages 30–35 years) and to guide management. The classification of the population in terms of risk is indeed the object of screening, and therefore, the longitudinal predictive value should be increasingly used to evaluate HPV testing as primary screening test.

At present, cocktail testing for 13 high-risk HPV types with Hybrid Capture 2 is the first clinically validated system and widely used in some countries. However, based upon more recent data showing the most aggressive natural history of HPV 16 and 18 as compared to the remaining high-risk HPV types, current discussions are considering the introduction of type-specific HPV tests in screening and clinical management (International Agency for Research on Cancer, 2005; Khan *et al*, 2005; Schiffman and Castle, 2005; Arbyn *et al*, 2006; Cuzick *et al*, 2006a).

## SUSTAINABLE SCREENING AND THE POLITICAL ACCEPTABILITY

The decades of the 1990s and 2000s have also witnessed trials on the use of low technology options to introduce screening in populations that lack the essential social and health structures required to make screening a success. In some instances, the lack of screening also reflects to the lack of political will to prioritise cancer prevention in women. These limitations are often presented as largely linked to the technology itself, but screening is a public health concept closely related to socioeconomic development, and therefore, changing technology does not guarantee a successful screening programme. For example, a number of observations in developing countries have shown that in addition to a very limited and socially selected screening coverage, a significant fraction of the failures refer to the inadequate follow-up of women with abnormal cytology. These are issues of country development, of availability and population access to medical facilities for diagnostics and treatment and, in more general terms, of sustainable health services and social equity (Denny *et al*, 2006).

Research on visual inspection methods (VIA), self-sampling strategies for HPV DNA, reduction of screening protocols to two-step (screen and treat) rather than three-step (screen, diagnose and treat), and development of lower cost, lower technology screening tests for HPV is ongoing, and results have already been reported. In Peru, comparisons were made between conventional cytology, liquid-based cytology, VIA and HPV testing (laboratories for HPV tests were out of the country). The study concluded that a strategy involving HPV testing as the primary screening test was the best option, provided that local infrastructure is developed and new HPV testing methods that result in lower cost are used (Almonte *et al*, 2007). In India, a VIA programme was effective in reducing the incidence of cervical cancer, of advanced cervical cancer and of mortality due to cervical cancer (Sankaranarayanan *et al*, 2007). Operational research should carefully consider strategies that would broaden the age ranges of the preventive interventions to accommodate logistics and health-care structures in place. For example, programmes could jointly offer HPV vaccine to adolescents (or to infants) and some form of screening to their mothers during the vaccination sessions already in place in most developing countries.

More recently, mathematical models are exploring alternative strategies that combine the power of vaccination to simplified screening schemes towards strategies that are sustainable in their context (Garnett *et al*, 2006; Raffle, 2007).

Several of such studies have been published for developed and developing countries, and estimates of the threshold costs of the vaccine are being produced to make them attractive to most populations. For example in Brazil, a cost of less than 25 international dollars per vaccinated girl aged less than 12 years of age followed by three screening rounds over lifetime in the intervals 35–45 would be considered very cost-effective under standard criteria (Goldie *et al*, 2007).

## VACCINATION OR SCREENING, VACCINATION AND SCREENING

It seems plausible that HPV vaccines will be, on the long run, the answer to cervical cancer and likely the answer to most of the other cancers of the female and male external genital tract. Once the extent of the cross protection is understood and/or additional HPV types are incorporated into the vaccines, the requirements for screening will be further reduced and eventually eliminated. In the interim, HPV vaccination should be made available to most women. The reasoning for it is several fold:

### Susceptibility by age

At any given point in time, the prevalence of HPV 16 and/or 18 among women participating in screening activities is low (less that 3% in most populations) and of women exposed to both 16 and 18 is less that 1%. The lifetime cumulative exposure to any of these two viral types is in the order of 20% in high-risk populations. Therefore, the vast majority of women remain susceptible to new infections throughout their sexually active life. Vaccines including HPV 6 and 11 will further increase their attraction for the prevention of genital warts (GWs) in sexually active women and possibly among men as well (Dunne *et al*, 2007).

### Efficacy by age

Human papillomavirus vaccines are efficacious in all age groups within the 15–26 years range. Although there are not at present individual studies reporting on HPV vaccine efficacy in sexually active adult after age 26 years, or broken down by age groups to investigate efficacy trends by age, the combined results from Phase III trials have shown anecdotal CIN 2+ cases in the vaccinated women (usually in the context of lesions with multiple HPV types), and, therefore, one could assume that protection is consistent in the 15–26 years range (Paavonen *et al*, 2007; The Future II Study Group, 2007). The Future II study has reported on an ‘intention-to-treat’ analysis in which women were included in the study irrespective of their HPV and cytology status at entry (except in the presence of HSIL requiring diagnostic and treatment). End points were CIN 2/3 cases related to one out of the four HPV types included in the vaccine (6, 11, 16 and 18) and were counted as from day 1 after first injection. A sizable proportion of women were HPV positive at entry (9% to HPV 16 and 4% to HPV 18) and/or had an abnormal cytology (ASCUS and LSIL combined 10.5%) both in the vaccinated and control groups. In these women, the efficacy of the vaccine was estimated at 44% (95% CI: 26–58). The equivalent efficacy estimates in the ‘per protocol’ populations was 98% (95% CI: 86–100). The reduction in the observed efficacy estimate should be interpreted as the absence of any therapeutic effect when HPV DNA-positive women are vaccinated rather than any putative-reduced biological response linked to age. This interpretation is consistent with the results showing no effect on HPV prognosis in women receiving the bivalent vaccine (Hildesheim *et al*, 2007). Ongoing trials should provide the necessary evidence on efficacy for women above the age of 26 years. Cost-effective analyses examining the impact of age at vaccination in the presence of variable underlying HPV prevalence rates by country and age should clarify in the near future the opportunities linked to vaccination of middle-aged sexually active women.

### Immunogenicity by age

The vaccines are immunogenic in a wide age range. Bridging studies have shown that vaccinating women in the age groups 26 years and above induces universal antibody response and, although the antibody titres are significantly lower as women age, they remain at a significantly higher level than the titres induced by natural infection; moreover, more indication of immune memory in the quadrivalent vaccine trial has been reported (Olsson *et al*, 2007). Although to date, no correlate of protection can be linked to antibody titres, largely from animal experiments it is generally accepted that antibody production and protection from infection are interrelated.

### Safety by age

Human papillomavirus vaccines are safe in the 15–26 age range and within the limits of 6–7 years of follow-up (http://www.who.int/vaccine_safety/en/; GACVS, 2007). There is so far no indication that safety concerns might arise in the elderly, but again, this is an issue to be answered by ongoing trials and Phase IV long-term follow-up on millions of persons.

Over the predicted fraction of cervical cancer cases unrelated to 16 or 18 that will continue to occur in vaccinated women, an important key consideration for the integration of HPV vaccines into existing screening efforts, including women up to 45–50 years is that these vaccines are not therapeutic (Hildesheim *et al*, 2007; The Future II Study Group, 2007). Therefore, when vaccinating adult women, it is important to make certain that any ongoing neoplastic process (women either persistent HPV positive or with prevalent lesions) is identified by adequate screening. However, ruling out underlying infection with the HPV types included in the vaccine is not a prerequisite for vaccination, since less that 1% of women would be excluded from vaccination based upon a positive test for HPV 16 and 18 DNA. It will also be important to explain to women the need to continue surveillance for cervical neoplasia after vaccination and that the promise of a 70% protection against cervical cancer only applies fully to HPV 16, 18-negative women at the time of vaccination.

### Continue screening, yes but how?

While cytology remains as the primary screening option, scientific evidence already available suggests that follow-up after HPV vaccination should drift over time towards a generalised adoption of the new generation screening protocols, largely based upon the detection (and typing) of HPV DNA. The interest of these new protocols, in which the advantages of an HPV test over cytology in terms of sensitivity and positive predictive value are fully recognised, is further enhanced by the understanding that after eliminating HPV 16 and 18 from the spectrum of HPV infections and related lesions in vaccinated adolescents, the performance of the cytology as screening test will be further reduced (Franco *et al*, 2006). In a context in which women are and will continue to be regularly followed up, one could argue that the screening test used (cytology or HPV tests) may not represent a significant alternative, other than the costs of the number of visits and the need of repeated examinations generated by each technology. However, current scientific evidence clearly recommends to use as a first-line screening test the most sensitive – HPV DNA testing – and to use the most specific – cytopathology – for triage and diagnosis. In many parts of the world, screening alternatives that require fewer visits are essential, and models calibrated to developed countries are being developed to guide public health decisions in defining the appropriate and cost-effective strategies for genital cancer prevention (Kim *et al*, 2005; Garnett *et al*, 2006; Elbasha *et al*, 2007).

On medical grounds, even if HPV vaccination would achieve a similar level of mortality reduction as currently achieved by organised screening programmes (i.e., close to 70%), primary prevention of cervical lesions has significant clinical and human advantages over secondary prevention, because it avoids most of the uncertainties and unwanted side effects of cytology-based screening programmes. These include the requirement of frequent and repeated visits of millions of women with a normal cervix, the 4–5% uncertain cytological results (ASCUS and others) requiring additional diagnostic protocols, the often required biopsies and the eventual surgical treatments for conditions that otherwise would regress spontaneously in a sizeable proportion (i.e., most of the CIN 2 cases). The latter, a direct consequence of some of the most recent classifications of cervical lesions (i.e., the Bethesda System), has repeatedly been linked to unwanted obstetrics consequences (Kyrgiou *et al*, 2006).

## THE IMPACT OF GENITAL WARTS IN THE SCREENING/VACCINATION SCENARIO AND THE PREVENTION OF OTHER GENITAL CANCERS

One of the two vaccines available includes HPV 6 and 11 antigens and has shown high efficacy against external GWs (Garland *et al*, 2007). The prevalence of GW has been estimated at 1% of the sexually adult population, is common in young persons and represents a significant burden to health services. The economic impact of GW is being evaluated, and it is anticipated that the quadrivalent vaccine will offer a significant advantage on these grounds, over and above, the cancer prevention potential (Elbasha *et al*, 2007). Finally, the quadrivalent vaccine has already shown high efficacy against the preneoplastic lesions of the vulva (VIN 2/3) and vagina (VAIN 2/3) related to HPV 16 and 18. At present, screening for these conditions is erratic and no organised activities are in place even in developed countries with centralised cervical cancer screening programmes. Therefore, prevention of these cancers represents a net significant advantage of current HPV vaccines over screening protocols. Results from the bivalent HPV vaccine have not yet been reported.

## THE CHALLENGE AND THE ORGANISATION

The tools and the momentum seem now ready to redesign novel strategies for cervical cancer prevention in developed and developing countries and populations. The axes of action seem to call for efforts in at least four directions:

### Research for intervention

Table 2 summarises some of the activities that are important to accelerate the successful introduction of HPV vaccines in the different scenarios in the world.

### Education

Both professional and public need to renew and increase awareness on the burden of disease and the new opportunities for prevention. Educational messages need to understand the complexities of a vaccine against a sexually transmitted infection, and the cultural specificities that have to be considered to ensure acceptability and high uptake.

### Financial support

Continue the work initiated by a number of institutions and funding agencies to ensure that support is being directed to make full use of these new opportunities. Cervical cancer might not be regarded as a social priority in some countries if compared to other vaccines or therapies for the prevention of more prevalent conditions such as malaria or AIDS. However, excellent HPV vaccines are the feasible option now and all efforts have to be made to integrate them into the vaccine delivery schemes.

### Coordination

The arrival of a new vaccine is a complex interdisciplinary exercise requiring different social abilities and expertise. Multiple partial initiatives are under way, and coherence in the messaging and programme development should be viewed as an advantage. This refers to the management of information, addressing research issues and deploying the network and the logistics to reach women in the world.

## CONCLUSION

The qualitative new protocols for cervical cancer prevention after the seminal work of Papanicolaou are now being written, taking full advantage of the HPV-based technology for screening and vaccination. The deployment of such protocols has the potential to complete Papanicolaou's goal of cervical cancer eradication by extending the benefits of prevention to the developing populations of the world.

## Figures and Tables

**Table 1:** Key results from phase III trials of HPV vaccines

**Vaccine name**	**Gardasil®**	**Cervarix®**
Time of follow-up	36 months (advanced)	15 months (interim)
HPV types included	6, 11, 16, 18	16, 18
*Efficacy HPV 16 or 18 CIN 2*+	Proven	Proven
Efficacy HPV 16 CIN 2+	Proven	Proven
Efficacy HPV 18 CIN 2+	Proven	Not yet proven^a^
Efficacy 16 or 18 CIN 2	Proven	Proven
Efficacy 16 or 18 CIN 3	Proven	Not yet proven^a^
Therapeutic efficacy	None	None
Efficacy on VIN 2/3	Proven	Not yet reported
Efficacy on VAIN 2/3	Proven	Not yet reported
Efficacy on genital warts	Proven	Not in target
Safety at 6 years follow-up	Safe^b^	Safe^c^
Tolerability	Acceptable	Acceptable
Cross protection (persistent HPV infection)	6 months	12 months
Cross protection (lesions)	Reported	Not yet reported
Duration of protection^d^	5–6 years	5–6 years
Immunogenicity in preadolescents	Proven	Proven
Immunogenicity in older women	Proven	Proven
Immune memory at 6 years	Proven	Not yet reported

CIN=cervical intraepithelial neoplasia; HPV=human papillomavirus.

a Proven in combined analysis of Phase II and III trials.

b In postlicensing evaluation (http://www.who.int/vaccine_safety/en/).

c In clinical trials.

d Corresponds to duration of trials in 2007.

**Table 2:** Selection of current research priorities to accelerate HPV vaccine arrival to high-risk countries for cervical and other genital cancers

• Continue generating estimates of the burden of HPV and related cancer. This is particularly important in developing areas, where health statistics are of limited completeness and are likely to underestimate the extend of the cancer burden in women
• Advance in the modelling exercises at a regional level to help estimate the incidence of cervical cancer from HPV surveys
• Complete trials of HPV vaccines in infants with a view to its incorporation into the existing vaccination programmes
• Complete trials of HPV vaccines in men. Studies on the potential negative impact of a gender-specific vaccination. Vaccine acceptability in different cultures should be completed
• Complete trials in the immunosuppressed to guide use of HPV vaccines and vaccine choice in countries with high prevalence of HIV and malaria
• Complete trials in women above the age of 26 years to estimate the full impact of strategies that include massive vaccination campaigns of women aged 9–45+ years
• Include in the Phase IV trial designs the evaluation and rationalisation of the catch-up strategies in adult women that will occur in developed countries
• Complete the evaluation of the impact of HPV-type-specific cross protection of current vaccines and continue research into polyvalent vaccines covering a wider spectrum of the cancer-causing HPV types
• Advance in the evaluation of sustainable alternatives for screening in developing countries
• Advance in the models for integration of HPV vaccines and in the definition of subsequent screening programmes for the surveillance of vaccinated women

HPV=human papillomavirus.
